# Supplementary material for: Tumorigenicity decrease in Bcl-xL deficient MDCK cells ensuring the safety for influenza vaccine production
Source: PLoS One. 2024 Dec 16;19(12):e0311069. doi: 10.1371/journal.pone.0311069 (PMC11649150; doi:10.1371/journal.pone.0311069)
Supplement: S1 File — (ZIP) [file pone.0311069.s002.zip › S2_File/3_KEGG_enrichment/scatter/union.DE_gene.subcluster1_KEGG_enrichment_scatter.pdf]

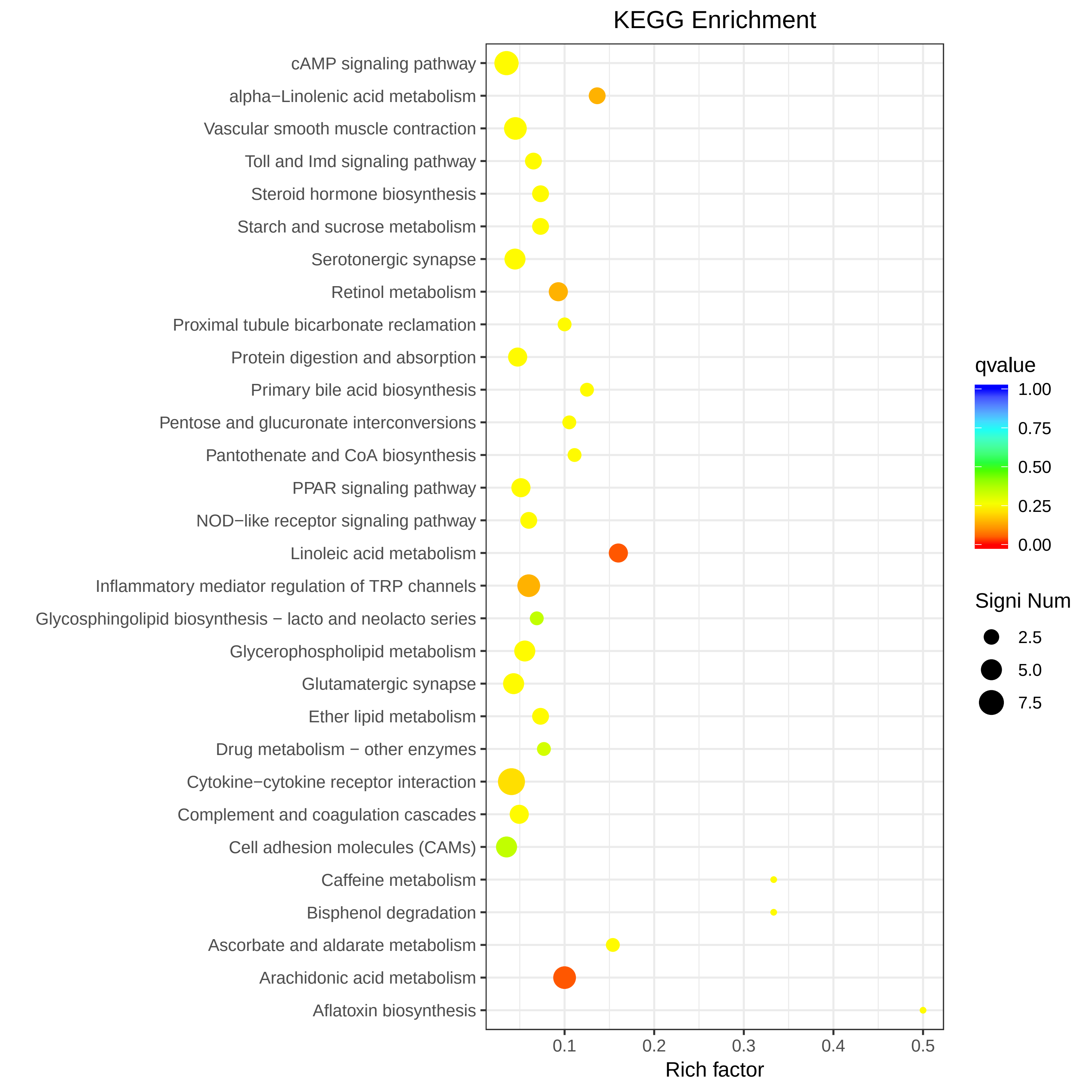
KEGG Enrichment
cAMP signaling pathway
alpha−Linolenic acid metabolism
Vascular smooth muscle contraction
Toll and Imd signaling pathway
Steroid hormone biosynthesis
Starch and sucrose metabolism
Serotonergic synapse
Retinol metabolism
Proximal tubule bicarbonate reclamation
Protein digestion and absorption
Primary bile acid biosynthesis
Pentose and glucuronate interconversions
Pantothenate and CoA biosynthesis
PPAR signaling pathway
NOD−like receptor signaling pathway
Linoleic acid metabolism
Inflammatory mediator regulation of TRP channels
Glycosphingolipid biosynthesis − lacto and neolacto series
Glycerophospholipid metabolism
Glutamatergic synapse
Ether lipid metabolism
Drug metabolism − other enzymes
Cytokine−cytokine receptor interaction
Complement and coagulation cascades
Cell adhesion molecules (CAMs)
Caffeine metabolism
Bisphenol degradation
Ascorbate and aldarate metabolism
Arachidonic acid metabolism
Aflatoxin biosynthesis
0.1
0.2
0.3
0.4
0.5
Rich factor
qvalue
1.00
0.75
0.50
0.25
0.00
Signi Num
2.5
5.0
7.5
